# Investigation of Thermal-Induced Changes in Molecular Order on Photopolymerization and Performance Properties of a Nematic Liquid-Crystal Diacrylate

**DOI:** 10.3390/ma15134605

**Published:** 2022-06-30

**Authors:** Qian Wang, Stephen T. Wellinghoff, Henry Ralph Rawls

**Affiliations:** 1 Division of Research, Department of Comprehensive Dentistry, University of Texas Health Science Center at San Antonio, San Antonio, TX 78229, USA; hrawls1135@gmail.com; 2 Core Facility Center for Life Sciences, University of Science and Technology of China, Hefei 230026, China; 3 Chemistry and Chemical Engineering Division, Southwest Research Institute, San Antonio, TX 78238, USA; stephen.wellinghoff@swri.org

**Keywords:** nematic, molecular order, photopolymerization, phase transition, polymerization rate, crosslink, glass transition temperature, shrinkage, dental application

## Abstract

Polymerization shrinkage and associated stresses are the main reasons for dental restorative failure. We developed a series of liquid crystal diacrylates and dimethacrylates which have markedly low polymerization shrinkage. In order to fully understand the effects of temperature-induced changes of molecular order on the photopolymerization process and performance properties of the generated polymers, the photopolymerization of a difunctional acrylate, 2-*t*-butyl-1,4-phenylene bis (4-(6-(acryloyloxy)hexyloxy)benzoate), which exists in the nematic liquid crystalline phase at room temperature, was investigated as a function of photopolymerization temperature over the nematic to isotropic range. Morphological studies suggested that a mesogenic phase was immediately formed in the polymer even if polymerization in thin films occurred above the nematic-to-isotropic (N→I) transition temperature of the monomer (*T_n-i_* = 45.8 °C). Dynamic mechanical analysis of 2 × 2 mm cross-section bar samples polymerized at 60 °C showed reduced elastic moduli, increased glass transition temperature and formation of a more crosslinked network, in comparison to polymers formed at lower polymerization temperatures. Fractography analysis showed that polymers generated from the nematic liquid crystalline phase underwent a different fracture pattern in comparison to those generated from the isotropic phase. Volumetric shrinkage (2.2%) found in polymer polymerized from the nematic liquid crystalline phase at room temperature was substantially less than the 6.0% observed in polymer polymerized from an initial isotropic phase at 60 °C, indicating that an organized monomer can greatly contribute to reducing cure shrinkage.

## 1. Introduction

Resin composites were originally introduced as a replacement for mercury-based alloys in the 1960s. Since then, these materials have undergone significant developments, and have become the primary choice in restorative dentistry due to advantages such as tooth-like appearance, low thermal conductivity, no mercury leakage and no galvanic currents. A modern dental composite system includes (1) a curable monomer matrix in which is dispersed (2) a high-volume percent of micro- or nano-sized inorganic fillers that are strongly bonded to the monomer matrix by (3) a coupling agent [1,2].

Despite the promising properties over amalgam, resin composites have a relative short life span with mean annual failure rates between 1% to 5%, with the failure risk increasing quickly in large posterior restorations [3,4,5]. Polymerization shrinkage and associated stresses are the main reasons for failure. Polymerization shrinkage, resulting from the decreased inter-molecular Van der Waals distance due to conversion of double bonds to single bonds, causes stress build-up within the bulky composite as well as between the tooth-composite interface. As the stresses are concentrated at weak points and eventually relieved by either adhesive or cohesive fracture, problems of marginal leakage, microbial attack, discoloration and secondary caries occur, that finally lead to failure of composite restorations [6,7]. In order to reduce polymerization-associated stress, dental professionals have developed an incremental layering technique [8] to reduce stress build-up in large, multi-surface restorations. They also modified the light activation protocol [9,10,11], such as using a pulse-photo mode or a soft start, to slow the early-stage polymerization process in attempting to prolong the period that the composite can flow to compensate for shrinkage. These techniques have achieved some success, but do not completely solve the problem [12]. Material scientists have adopted new fillers that can be added to a higher percentage in composites to reduce shrinkage [1], and low-modulus adhesives or coupling agents that can generate elastic deformation during polymerization to relieve stress [13]. Unfortunately, high content of fillers usually decreases flowability which is critical for relief of polymerization shrinkage, and low-modulus interface may become the mechanically weak points which threaten the long term performance of composites. Therefore, it is important to develop low shrinkage resins to reduce polymerization shrinkage and associated stressed for restorative composites.

Researchers have reported several strategies to design and synthesize low shrinkage dental resins. One strategy is to reduce double bond concentration by functionalizing into dimethacrylates with bulky rings [14,15] or adding dimer molecules [16]. However, these monomers generate less crosslinked polymer matrix, which may cause concern for long-term stability. Another strategy is to break bonds by opening rings during polymerization, using the rationale that the increased length between broken bonds can compensate for double bonds converting to single bonds. These ring-opening monomers include spiro orthocarbonates [17,18] and siloranes [19], in which a silorane-based composite has been used in a commercial product. Our group proposed the strategy of structural expansion to compensate for polymerization shrinkage using liquid crystal monomers, based on the rationale that the transformation from ordered monomer to amorphous polymer generates sufficient volume expansion to reduce overall shrinkage [20]. The inherent order of monomers in the liquid crystalline phase may significantly alter the mechanisms of polymerization and affect the generated polymer networks. Increased polymerization rate [21,22,23,24,25] and reduced shrinkage [20,26,27,28,29] have been observed in monomers polymerized in the nematic phase. In addition, tensile modulus is enhanced along the oriented polymer networks [30,31,32] and fracture toughness is improved if highly ordered smectic domains are formed during polymerization [33,34].These advantages of liquid crystal monomers are highly desirable in dental applications. Among the three main distinct classes of liquid crystals, the rod-like nematic monomers are particularly suited for dental applications, mainly due to inherently low viscosity and liquid-like mobility [35].

We developed a series of nematic diacrylate/dimethacrylate monomers for which nematic-to-isotropic (N→I) phase transition temperatures range from 40–140 °C. Monomers with higher N→I transition temperatures are assumed to align with a higher molecular order at oral temperatures and therefore, may produce more structure-related advantages as dental restorative resin candidates, such as lower shrinkage. Meanwhile, we find that such monomers are meta-stable in the nematic state and tend to crystallize during storage. The probability of phase change and crystallization increases as the N→I transition temperature increases and is exacerbated in the presence of reinforcing mineral fillers. In order to be used in commercial applications, it is important for such liquid crystal monomers to reach a balance between maintaining phase stability during storage and structure-related property improvements. Our empirical experience is that, in some cases, this may be accomplished by blending of individual monomers.

**Figure 1:**
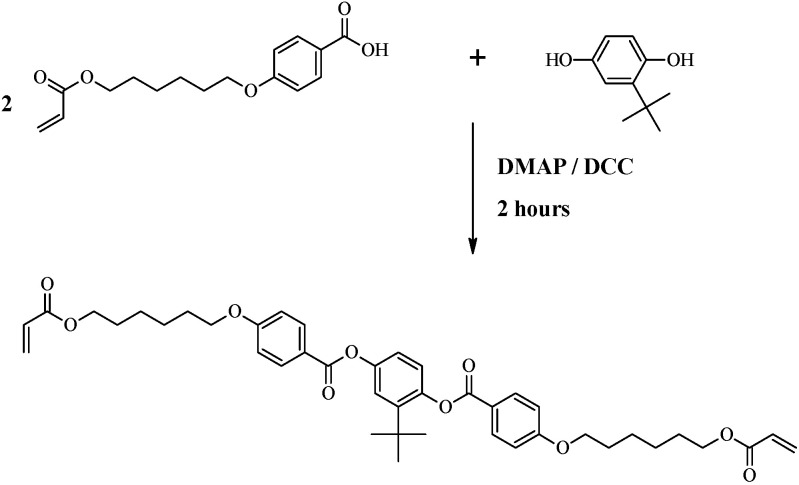
Synthesis of the nematic monomer 2-*t*-butyl-1,4-phenylene bis (4-(6-(acryloyloxy)hexyloxy)benzoate) (tBC6DA).

In order to maximally benefit from the nematic phase of the liquid crystal monomers, it is necessary to fully understand the effects of molecular order on the photopolymerization process and performance properties of the generated polymers. In this report, we focused on thermal-induced changes in the molecular order. A nematic monomer 2-*t*-butyl-1,4-phenylene bis (4-(6-(acryloyloxy)hexyloxy)benzoate) (abbreviated tBC6DA) (Figure 1) was photopolymerized at temperatures that spanned from the nematic to the isotropic range, similar to conditions encountered in dental practice. Performance properties, including the polymerization rate, which was measured by degree of double bond conversion, static and dynamic mechanical properties and shrinkage were evaluated. This experimental design was expected to simulate the situation that liquid crystal monomers with different N→I transition temperatures polymerized at the same oral temperature. We expected the results would provide valuable information for future liquid crystal monomer synthesis and formulation.

## 2. Materials and Methods

Liquid crystal monomer tBC6DA was synthesized and purified in our lab. All purchased materials were used without further purification. The molecular structure of tBC6DA was characterized using ^1^H nuclear magnetic resonance (NMR), and the purity was analyzed using high pressure liquid chromatography (HPLC). Photopolymerization at temperatures ranging from the isotropic to the nematic state was studied in both thin film (ca. 100 microns) and 2 × 2 × 20 mm bar format. In the former case, polarized optical microscopy was used to study the morphology and Fourier transform infrared (FTIR) was used to measure the degree of double bonds conversion at different photopolymerization times and temperatures. In the latter case, fracture strength/morphology, dynamic mechanical properties and shrinkage were measured under similar conditions, albeit with an extensive photo-postcure to complete double bond conversion to >90%.

### 2.1. Materials

*t*-butyl hydroquinone, 4-dimethylaminopyridine, dicyclohexyl dimethylcarbodiimide (1M solution in dichloromethane), camphorquinone, *N*,*N*-dimethylamino ethylmethacrylate, anhydrous dichloromethane and acetone were purchased from Sigma-Aldrich (St. Louis, MO, USA). Hydrochloric acid, sodium chloride, sodium bicarbonate and sodium sulfate were purchased from EMD Chemicals USA (Gibbstown, NJ, USA). Finally, 4-(6-acryloyloxyhexyl-1-oxy) benzoic acid was prepared as previously reported by Portugall et al. [36].

### 2.2. tBC6DA Monomer Synthesis and Characterization

The synthesis was a modification of a procedure reported previously [28], using a catalyst of 4-dimethylaminopyridine coupled with an esterification-promoting agent dicyclohexyl dimethylcarbodiimide (Figure 1). The entire reaction was carried out in an inert atmosphere of nitrogen. In total, 0.034 mol 4-(6-acryloyloxyhexyl-1-oxy) benzoic acid, 0.015 mol *t*-butyl hydroquinone and 0.003 mol dimethylaminopyridine were slurried in 50 mL anhydrous dichloromethane. The mixture was chilled to 0 °C in ice water, and 0.041 mol dicyclohexyl dimethylcarbodiimide was added. The reactants were then warmed to room temperature and stirred for 2 h. After filtering out solids, the solution was washed with 0.5 N hydrochloric acid, saturated sodium bicarbonate and saturated sodium chloride solution. After drying over anhydrous sodium sulfate, and roto-evaporating to remove most of dichloromethane, the crude compound was further purified with column chromatography on silica gel (Alfa Aesar 60, 230–400 mesh) using a mixture of 98.5% dichloromethane and 1.5% acetone as the eluent. After removal of the solvent by vacuum evaporation, white crystals were formed at a yield = 54%. This sample was subsequently analyzed by HPLC and ^1^H NMR spectroscopy in CDCl_3_ solvent using tetramethylsilane as an internal standard (Varian INOVA 400 spectrophotomer, 400 Hz). 

### 2.3. Photopolymerization of tBC6DA Monomer

Monomer tBC6DA was mixed with 0.6% (*w*/*w*) camphorquinone (QC) photoinitiator (absorption band: ca. 400–500 nm, peak 470 nm) and 1.4% (*w*/*w*) *N*,*N*-dimethylamino ethylmethacrylate (DMAEMA) photoactivator to form a photopolymerizable mixture. The camphorquinone was dissolved in DMAEMA prior to mixing with tBC6DA. 

#### 2.3.1. Characterization of Temperature and Polymerization Induced Phase Change

Birefringent texture and polymerization-induced phase transition/segregation were monitored through cross-polars on an Axioskop 2 microscope (Carl Zeiss MicroImaging, Inc., Thornwood, NY, USA). Polymerization temperature was digitally controlled by a Peltier heated stage (Instec, Boulder, CO, USA). In order to determine polymerization-induced phase transition, a thin film (ca. 100-micron thickness) of the mixture between glass plates was continuously exposed to an Optilux 400 blue-light curing lamp (ca. 440–500 nm emission band, peak at 460 nm; integrated irradiance over wavelength distribution = 400 mW/cm^2^) over a period of up to 90 s and continuously monitored.

#### 2.3.2. Determination of Degree of Conversion

The degrees of conversion in thin ca. 100 micron thick films deposited on a ZnSe crystal were studied using a hot-stage FTIR spectrometer (Bruker Vector 22, Billerica, MA, USA) in a single reflection attenuated total reflection (ATR) configuration. While exposed to an Optilux 400 blue-light curing lamp, which was fixed 5 cm from the monomer, the spectrum of photoinitiated monomer was recorded every 10 s for the first 60 s, then every 30 s from 60 to 180 s, and then every 60 s up to a total of 300 s at predetermined temperatures. The degree of conversion was determined by comparison of the area change of the aliphatic C=C peak (approximately 1635 cm^−1^), referenced to the aromatic C=C peak (approximately 1604 cm^−1^) internal standard since its absorption did not change with extent of polymerization [37]. 

### 2.4. Characterization of Mechanical and Thermomechanical Properties of Polymers

In order to determine mechanical and dynamic mechanical properties and measure polymerization-induced shrinkage, bar-shaped polymer specimens were made in 2 × 2 × 25 (mm) rectangular glass tubes. Monomer containing the photoinitiation system was placed in a one-end sealed glass tube, heated to just over melting temperature (*T_m_*), and then cooled in an oil-bath to the predetermined polymerization temperatures bridging the nematic to isotropic phase range: room temperature (RT), 40, 50 and 60 °C. The sealing of the monomer in the tube minimized the potential for oxygen inhibition during polymerization. In order to promote maximal conversion of double bonds, polymerization was carried out in two steps: 3 min in the oil bath under an Optilux 400 blue-light curing lamp at a flux of 400 mW/cm^2^ and then 10 min post cure in a halogen quartz lamp in an oven (CureLite plus™ Jeneric/Pentron, Inc., Wallingford, CT, USA, filtered broad spectrum emission 400 nm–520 nm, estimated flux = 500 mW/cm^2^) in order to drive the cure to >90% (by FTIR) prior to measuring the final shrinkage and mechanical properties. 

After removing the cured specimens from the glass enclosure, mechanical properties were determined by stressing cured bar specimens (2 × 2 mm cross-section) in a 3-point bending mode (20 mm span) using a variable strain mechanical tester (Instron 1125, Instron Corp., Norwood, MA, USA). Fractured surfaces of each sample were further observed using an Axioskop 2 microscope (Carl Zeiss MicroImaging, Inc., Thornwood, NY, USA) and scanning electron microscopy (LEO 435 VP, LEO Electron Microscopy, Cambridge, UK).

Thermomechanical response of the same samples was studied using dynamic mechanical analysis (Perkin-Elmer DMA 7, Waltham, MA, USA) in small displacement, three point bending mode. Photopolymerized samples, P(tBC6DA), were scanned from 10 to 160 °C, at a frequency of 1 Hz, a static force of 110 mN and a dynamic force of 100 mN, with a nitrogen purge rate of 20 mL/min. Elastic modulus (E′) and loss tangent (tan δ) were recorded as functions of temperatures. The glass transition temperature *T_g_* was determined as the maximum of the tan δ curve.

### 2.5. Determination of Polymerization Shrinkage

Densities of tBC6DA monomer and P(tBC6DA) polymers were measured with a density gradient column (DC-4, Techne Inc., Burlington, NJ, USA) for polymers cured within the tubes at several initial temperatures. Shrinkages were calculated from the density changes before (*ρ_m_*) and after polymerization (*ρ_p_*) for three specimens polymerized under the same conditions at each starting temperature.

$$shrinkage\left(\%\right)=100\times \left(\rho_{p}-\rho_{m}\right)/\rho_{p}$$

## 3. Results and Discussion

### 3.1. tBC6DA Monomer Characterization

The chemical structure was confirmed by NMR. ^1^H NMR (CDCl_3_, chemical shift-ppm relative to TMS standard): 8.16 ppm [m, 4H, 2×-O-phenyl (2, 6-H)-COO]; 7.0 ppm [m, 4H, 2×-O-phenyl (3, 5-H)-COO]; 7.12 ppm [m, 2H, -COO-(2-C_4_H_9_)-phenyl (5, 6 H)-COO)]; 7.24 ppm [s, 1H, -COO-(2-C_4_H_9_)-phenyl (3-H)-COO)]; 4.2 ppm [t, 4H, 2×phenyl-O-CH_2_-(CH_2_)_4_-]; 4.05 ppm [t, 4H, 2×-O-CH_2_-(CH_2_)_4_- CH_2_-O]; 6.42 ppm [d, 2H, 2×CH_2_=CH-COO]; 5.81 ppm [d, 2H, 2×H_2_-CH=CH-COO-(*trans*)]; 6.12 ppm [d, 2H, 2×H_2_-CH=CH-COO-(*cis*)]; 1.75 ppm and 1.85 ppm [both m, 4H+4H, 2×-O-CH_2_-CH_2_-(CH_2_)_2_-CH_2_-CH_2_-O-], 1.55 ppm [m, 8H, 2×-O-(CH_2_)_2_-CH_2_-CH_2_-(CH_2_)_2_-O-];1.38 ppm [s, 9H, C(CH_3_)_3_-]. Multiplet (m), doublet (d), singlet (s), triplet (t).

After melting the crystalline monomer at 72.0 °C (*T_m_*) and cooling, the nematic to isotropic transition temperature (*T_n-i_*) upon reheating is 45.8 °C during heating and 43.3 °C during cooling as measured by differential scanning calorimetry (DSC) (see Supplementary Materials, Figure S1) in close agreement with previous work on the same monomer produced using a different synthetic method [28].

### 3.2. Polymerization-Induced Phase Transitions 

**Figure 2:**
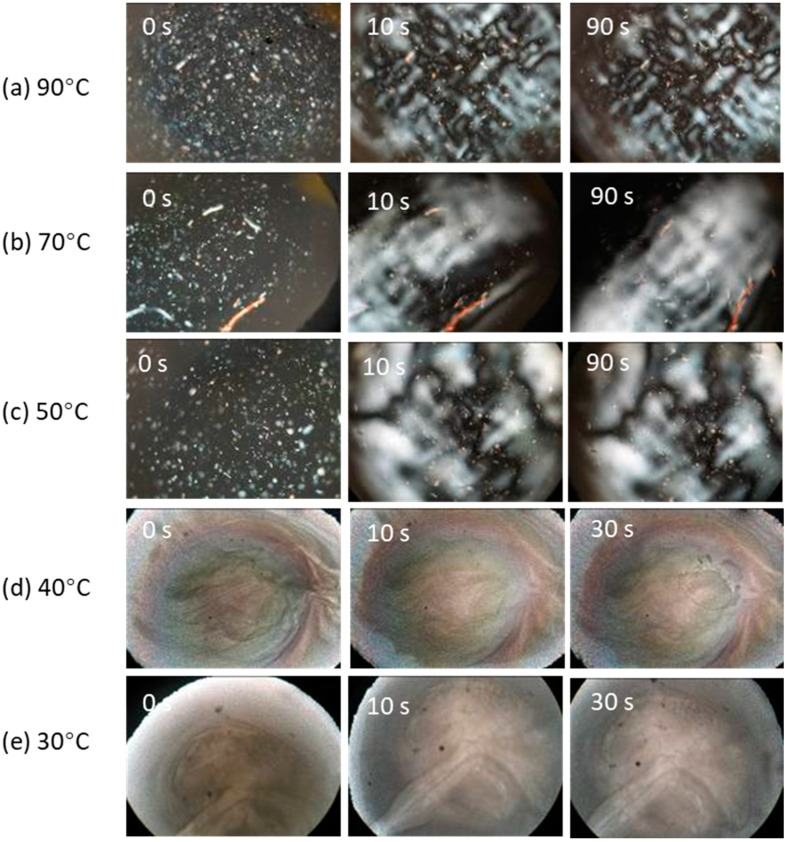
Polymerization-induced phase change/separation at temperatures from the nematic to the isotropic range. (**a**–**c**) polymerization started from the isotropic state, and (**d**,**e**) polymerization started from the nematic state. Magnification 100×.

Polymerization-induced phase transitions were divided into two groups based on the initial state of the monomer, as shown in Figure 2. When polymerization occurred in the nematic phase (at 30 °C and 40 °C, Figure 2d,e), the monomer/polymer mixture remained anisotropic and there were no detectable macro-scale morphological changes as photoexposure time increased. On the other hand, when polymerization occurred in the isotropic phase (50 °C and above, Figure 2a–c), a birefringent region was observed to separate from the isotropic phase after several seconds of photoexposure. The birefringent region is assumed to represent mesogenic polymer. When a liquid crystal monomer converts to a polymer, the mesogenic-to-isotropic transition temperature increases as the chain length grows [38,39]. Once the oligomer *T_n-i_* exceeds the polymerization temperature, the oligomer will phase separate to form a nematic and isotropic phase. The proportion of mesogenic phase appears to decrease as polymerization temperature increases (Figure 2). Of some interest is that the birefringent morphology appears to not change much between 10 s and 90 s of photoexposure. This segregation of mesogenic regions (larger bright regions), which occurs in the early stages of polymerization (10 s), is expected to have significant effects on polymerization behavior and structure-related polymer properties.

A feature worth commenting upon in Figure 2 is the dispersion of small birefringent regions that are apparent at 0 s in all the initially isotropic samples which did not change position, number or orientation as the photopolymerization proceeded. The same morphologies could have been present in the nematic phase at 0 second but their presence was not apparent because of the background birefringence of the nematic phase.

The melting point of these objects was >90 °C which suggested that they might be QC crystals (*T_m_* = 199 °C) that precipitated after the DMAEMA co-initiator/solvent had dissolved in the tBC6DA matrix prior to the photoexposure measurement. Although it has been generally assumed that a concentration of ca. 0.6% (*w*/*w*) QC is completely soluble in dental monomers, this might not be the case for tBC6DA. Since the tBC6DA was column purified and recrystallized (+99% by HPLC), there does not appear to be another obvious possibility. The phase transition of the pure tBC6DA that generated an isotropic area without birefringent regions also supported our assumptions (see Supplementary Information, Video S1). However, some significant amount of QC must have remained dissolved since photopolymerization did occur.

### 3.3. Degree of Conversion

**Figure 3:**
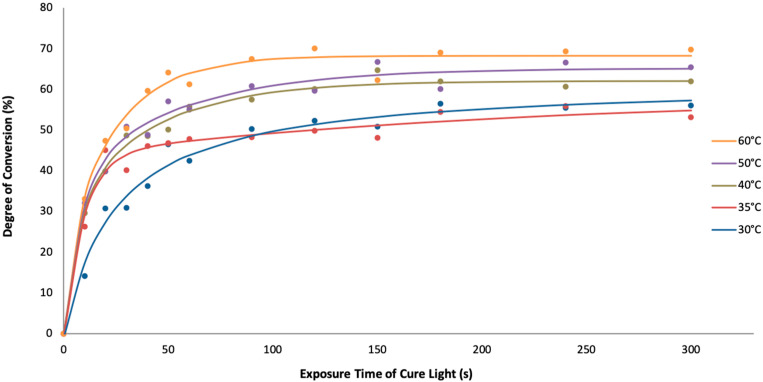
Conversion vs. exposure time and temperature range, bridging the nematic to the isotropic phase, in thin films similar to those used for the optical microscopy measurements in Figure 2.

As shown in Figure 3, aliphatic C=C bond conversion approached a plateau significantly less than 100% in all thin film samples (30–60 °C) at cure times of 300 s. The sample polymerized at 30 °C approached the same conversion plateau as the 35 °C sample but more slowly. Conversion decreased as polymerization temperature decreased from the isotropic to the nematic state. This conflicts with previous reports that both degree of conversion and polymerization rate are higher when starting in the nematic monomer state as compared to the isotropic state due to the more proximal positioning of reacting alkene groups in neighboring monomers in the former [24,40]. 

The increased ultimate degrees of conversion at 90 s with increased polymerization temperature in the thin films in Figure 3 might be explained both by a longer time for glass transition onset and temporary local excess volume generated during polymerization. If the polymerization rate is faster than the rate of overall volume shrinkage that develops when double bonds are converted to single bonds, the polymerizing material is not in volumetric equilibrium and a temporary excess of free volume is locally available [41], allowing higher degrees of conversion to be reached. 

In this regard, a later experiment which adopted with the same formulation/photoexposure, and thin film setup as used in Figure 3, showed that conversion vs. time curve of sample polymerized at room temperature (approximately 26 °C) was similar to that shown for the 60 °C sample in Figure 3. One possible explanation for this result is that in the experiment shown in Figure 3, the QC had partially crystallized (Figure 2) to different extents at each polymerization temperature, whereas in the later experiment, the QC had remained dissolved in the tBC6DA matrix at a higher concentration. In addition, in the later experiment, when the film was photoexposed for 780 s at room temperature at the same light flux, no C=C vibration at 1635 cm^−1^ could be detected, thus implying a conversion > 90%. This observation points out the importance of carefully determining the phase structure of the blend prior to photoexposure and the details of the storage and handling conditions (time and temperature) prior to the experiment.

### 3.4. Mechanical Properties and Fracture Analysis

Photopolymerization is an exothermic reaction, and therefore, if the heat of polymerization is not well dissipated, it will have important effects on a polymer structure, particularly when polymerization occurs at a temperature below but close to *T_n-i_*. Thus, the morphology present in 2 mm thick bar samples which were also postcured could be significantly different from that observed in thin film samples (ca. 100-micron thickness) used for cross-polarized optical microscopy and FTIR where the heat of polymerization is easily dissipated into the thermally conductive substrate. For example, when the initially rather opaque tBC6DA was cured in a rectangular mold at 40 °C, slightly below *T_n-i_*, its transparency increased, perhaps indicating that a more uniform structure was being produced. When polymerization begins at RT (approximately 26 °C) in the same mold, a change in overall opacity was not noticed.

**Figure 4:**
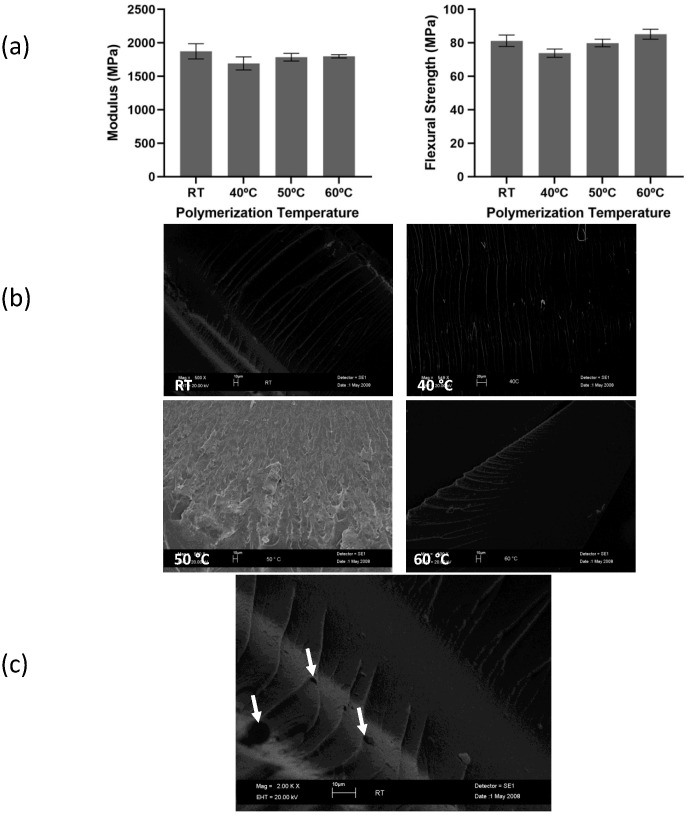
Mechanical properties and fracture surfaces of P(tBC6DA). (**a**) modulus (left) and flexural strength (right) of P(tBC6DA), (**b**) SEM fracture surfaces of P(tBC6DA) at different test temperatures, (**c**) higher magnification fracture surface of P(tBC6DA-RT) under SEM detailing void structure (small arrows) and general direction of crack growth toward the branching direction of the river lines (long arrow) at border between widely separated/narrowly separated river pattern. Data are presented as mean with SD.

The Young’s modulus and flexural strength of P(tBC6DA) are shown in Figure 4a. P(tBC6DA-RT) and P(tBC6DA-60 °C) showed higher moduli and flexural strengths with respect to P(tBC6DA-40 °C) and P(tBC6DA-50 °C). However, the difference between the highest and lowest values is small. Postulating any explanation for these differences is not possible in the absence of more through thickness morphological and spectroscopic information. In this regard, it should also be noted that that the bar samples were photocured in a different fashion from the thin film samples shown in Figure 2 and Figure 3 and cure could be different throughout the thickness of the bar even though the outer surfaces were protected from oxygen inhibition by the initial contact with the encapsulating glass. 

The fracture surfaces at RT and 40 °C (Figure 4b) show a clear “river-line” pattern under microscopic examination whose long dimension is oriented parallel to the large scale, collective crack growth direction [42]. The river lines represent the edge boundary of smaller scale cracks growing between the river lines. Depending on the rate of crack growth and the local stress state the small cracks can further bifurcate into cracks of smaller lateral dimension perpendicular to the river line long dimension. The appearance of cavities along the river-lines (small arrows in Figure 4c) indicates that lines are often separated by growth and coalescence of elongated cavities which further affect the overall crack growth. Overall, river line patterns are indicative of a brittle crack growth regime. 

At temperatures closer to and above the initial *T_n-__I_*, the crack surface morphology changes substantially (Figure 4b, 50 °C and 60 °C) in that the river lines are no longer present. Close inspection of the 50 °C fracture surface suggests more of a delocalized crack growth possibly indicating a greater contribution from ductile failure. The transition is complete at 60 °C where no fine river structure is seen on the crack face. 

### 3.5. Dynamic Mechanical Responses

**Figure 5:**
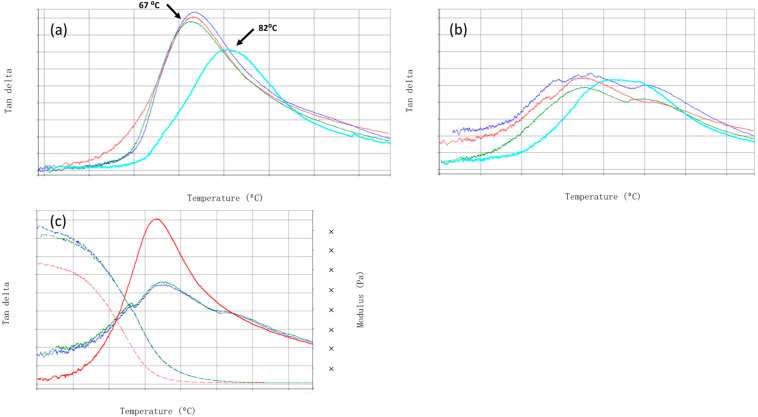
Dynamic mechanical responses (1 Hz in three point bending) of P(tBC6DA). Tan δ vs. temperature of the first scan (**a**) and the second scan (**b**). Arrows point to potential low and high temperature T*_g_* s. Samples polymerized at different temperatures are labeled with colors of RT (red), 40 °C (dark blue), 50 °C (green), 60 °C (light blue), (**c**) Tan δ and modulus vs. temperature for P(tBC6DA-RT), first scan (red), second scan (blue), third scan (green).

Glass transition temperatures of P(tBC6DA)s are shown in Figure 5a as the peak in the tan δ = E″/E′ where E″ is the loss modulus and E′ is the in phase storage modulus. All polymer samples show a *T_g_* in the first scan close to 67 °C except P(tBC6DA-60 °C), which has a *T_g_* of 82 °C. The second scan suggests that there are multiple *T_g_* s: for RT and 40 °C polymerized samples (ca. 55 °C, 70 °C, 110 °C), for 50 °C (ca. 70 °C, 110 °C) and for 60 °C (ca. 84 °C, 95 °C) (Figure 5b). Subsequent scans are identical to the second scan indicating that the morphology is fully developed after the first heating to 150 °C, well above the *T_g_* s of the first scan (Figure 5c). 

Further study would be required to determine the structure property relationship inherent in the ultimate development of three separate phases in P(tBC6DA-40 °C and RT) and two separate phases in P(tBC6DA-50 °C and 60 °C) after the first scan. Whether further completion of the polymerization and/or additional development of the initially metastable phase structure is responsible for these characteristics is unknown. Although the further maturation of the phase structure upon heating probably is not important for situ photo-polymerized dental restoratives carried out at oral temperatures, this process could be important for 3D photo printing applications where initial photocures are often followed up by thermal postcures.

**Figure 6:**
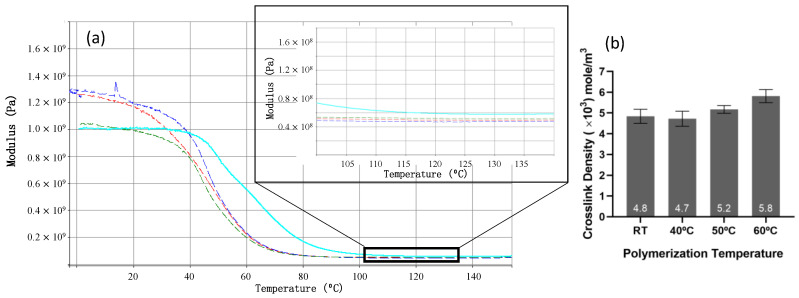
(**a**) Elastic storage modulus (E′) vs. temperature (first scan, 1 Hz), insert: magnified rubber plateau, samples polymerized at RT (red), 40 °C (blue), 50 °C (green), 60 °C (light blue), (**b**) calculated crosslink density. Data are presented as mean with SD.

The theoretical crosslink density (Figure 6b) calculated from the first scan, rubbery storage modulus of P(tBC6DA-60 °C) (Figure 6a) [43], is significantly higher when compared to samples polymerized at lower temperatures. This is of course consistent with the observation that the P(tBC6DA-60 °C) has a significantly higher *T_g_*. 

Also of interest is that the low strain, glassy storage moduli (E′ = ca. 1.0 GPa) of the P(tBC6DA-60 °C, 50 °C) photopolymerized in the initially isotropic state in three point bending are significantly lower than the P(tBC6DA-RT, 40 °C) (E′ = 1.3 GPa) samples photopolymerized in the initially nematic state (Figure 6a) even though the elastic modulus measured in three point bending (constant strain rate) in Figure 4a is only significantly higher (E = 1.8 GPa) for P(tBC6DA-RT). Ideally E (constant strain rate) = E′ (cyclic small strain in DMA) but this relationship can be modified by the specifics of stress vs. strain slope measurement.

### 3.6. Polymerization Shrinkage

**Figure 7:**
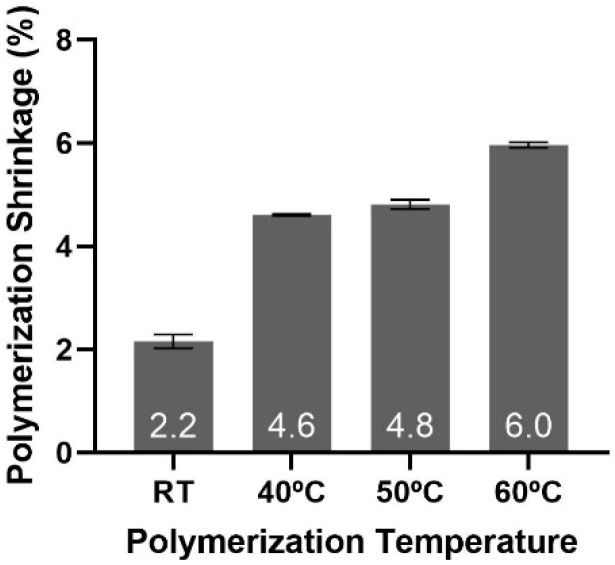
Polymerization shrinkage as a function of initial polymerization temperature. Data are presented as mean with SD.

Polymerization shrinkage in the 2 mm thick bar samples increases as the initial polymerization temperature increases (Figure 7). The lowest volumetric shrinkage, 2.2% for P(tBC6DA-RT), might be attributed to volume expansion generated when the nematic monomer converts locally to a more isotropic polymer or a more distorted nematic phase. Again, the detailed morphology or degree of polymerization was not measured as a function of depth in the bar which could have been different depending upon the local dissipation of the heat of polymerization or penetration of the photocure light. These factors could be of increased importance since the polymerization temperature is so close to *T_n-i_*.

## 4. Conclusions

The photopolymerization of the nematic monomer tBC6DA was first investigated in thin films at varying temperatures both below (30 °C, 40 °C) and above (50 °C, 70 °C, 90 °C) the monomer *T_n-i_* = 45.8 °C. At the higher temperatures, diffuse mesogenic regions phase separated from the initially isotropic regions as the polymerization proceeded. At the lower temperatures, the film remained in the nematic state during polymerization. However, small birefringent species were found in the initially highly purified and recrystallized tBC6DA (*T_m_* = 72 °C) prior to polymerization even at 90 °C, which were suspected to be the high melting point QC photoinitiator (*T_m_* = 199 °C). There was also disagreement between the degree of double bond conversion at 300 s photoexposure in thin film samples for the same polymerization conditions that might have been related to differing extents of crystallization of the QC component during storage of the sample after initial mixing of the initiator and co-initiator and tBC6DA. This suggests that the time-temperature solubility profile of the photoinitiator must be carefully assessed in new monomers.

The 2 mm thick bar samples, more completely photopolymerized at RT, 40 °C and 50 °C, 60 °C, showed differing behavior, in that samples polymerized at the two lower temperatures (<*T_n-i_* for monomer) showed a significantly higher sub *T_g_* E′ than the samples polymerized at higher temperature. In addition, the 60 °C sample had a higher post photopolymerized *T_g_* = 82 °C than the samples photopolymerized at a lower temperature (*T_g_* = 67 °C). This single phase structure was metastable to heating above the initial *T_g_* in that multiple *T_g_* s were observed subsequently. Finally, a considerably lower polymerization shrinkage of only 2.2% (*v*/*v*) was noted for the sample photopolymerized at RT compared to samples photopolymerized at higher temperatures, thus confirming the desirability of initiating photopolymerization deep within the nematic state. 

Unfortunately, tBC6DA is able to remain in the nematic state for only a few hours at room temperature before spontaneous crystallization. Thus, it is reasonable to expect that single component nematic monomers with similar chemical structures will not be sufficiently stable in the nematic state to provide acceptable storage stability for commercial performance. In separate studies, blending nematic monomers with certain comonomers and other compounds were observed to slow or prevent crystallization [44], thus demonstrating the feasibility of using high-nematic-transition-temperature monomers.

In addition, in order to generate both increased fracture strength and reduced polymerization shrinkage at high conversions, it is necessary to utilize monomer mixtures which can be polymerized from the nematic monomer into a lower density nematic polymer to compensate for shrinkage due to double bond conversion. Thus, photopolymerization process and performance properties have also been reported for a series of nematic monomer/dimer mixtures that exhibit high fracture toughness and low polymerization shrinkage at high conversions. Composites with these polymer blends have also been studied [45]. 

In summary, even though more understanding of the structure/properties of liquid crystal monomers has been shown for potential dental restorative applications in this paper, several issues must be addressed before commercialization can occur in an already crowded and rather entrenched marketplace that also includes building of 3D structures using digital light processing or stereolithography; these include: (1) considerably reducing the competitive synthesis cost of each monomer, preferably using the less cytotoxic methacrylate derivatives, subject to regulatory approval, (2) optimizing the mixture compositions of a minimal number of different pure monomers to produce a storage stable phase structure that can maintain a nematic state at the application polymerization temperature and at a sufficiently low viscosity for downstream processing and use and (3) optimizing the photoinitiator/stabilizer composition and mixing process in these downselected mixtures to achieve highly reproducible results (high fracture strength and low polymerization shrinkage at high conversions) under known light flux/temperature, preferably without the necessity of long post cures. 

## Supplementary Materials

The following supporting information can be downloaded at: https://www.mdpi.com/article/10.3390/ma15134605/s1, Figure S1: Phase transition temperatures of tBC6DA measured using DSC. Video S1: The phase transition of tBC6DA monomer at temperatures from the nematic to isotropic range.
