# Supplementary figures and images for: Investigation of Thermal-Induced Changes in Molecular Order on Photopolymerization and Performance Properties of a Nematic Liquid-Crystal Diacrylate
Source: Materials (Basel). 2022 Jun 30;15(13):4605. doi: 10.3390/ma15134605 (PMC9267439; doi:10.3390/ma15134605)

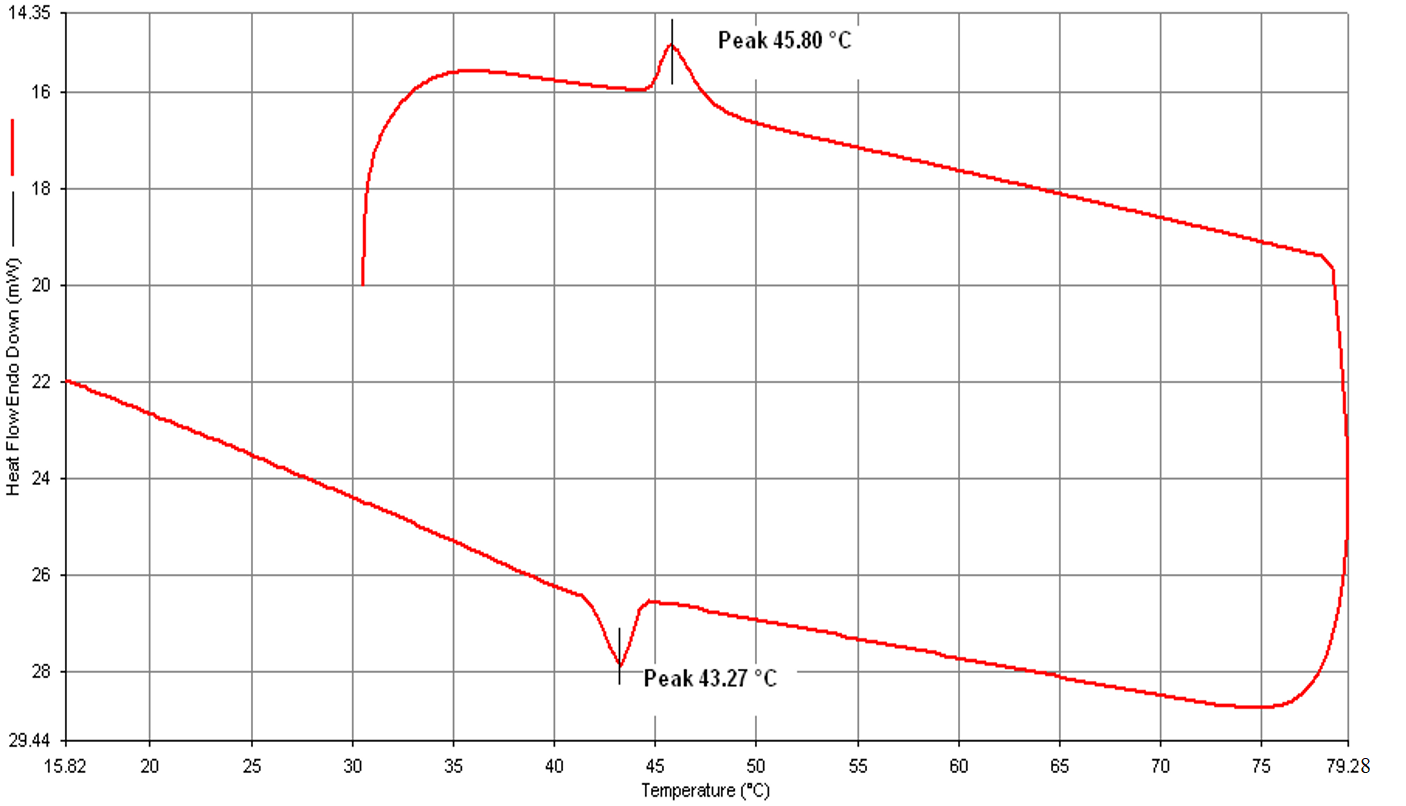

Supplement: Supplementary file 1 [file materials-15-04605-s001.zip › Figure S1.png]
